# Acknowledgment to Reviewers

**DOI:** 10.1080/15438600490436160

**Published:** 2004


					Experimental Diab. Res., 5:97, 2004
Copyright c
 Taylor and Francis Inc.
ISSN: 1543-8600 print / 1543-8619 online
DOI: 10.1080/15438600490436160
Acknowledgment to Reviewers
The Editors-in-Chief are grateful to the following colleagues for their careful evaluation of manuscripts submitted to Experi-
mental Diabesity Research during the year 2003:
John Baynes, Columbia, SC, USA
Frank Brosius, Ann Arbor, MI, USA
Michael Brownlee, New York, NY, USA
Chuck Burant, Ann Arbor, MI, USA
Norman Cameron, Aberdeen, Scotland
Subrata Chakrabarti, London, ONT., Canada
Mary Cotter, Aberdeen, Scotland
Amy Davidoff, Biddeford, ME, USA
Marc Donath, Zurich, Switzerland
Shimon Efrat, Tel Aviv, Israel
George Eisenbarth, Denver, CO, USA
Konrad Federlin, Giessen, Germany
Eva Feldman, Ann Arbor, MI, USA
Miyuki Furusho, Setagaya, Japan
Todd Leff, Detroit, MI, USA
Ake Lernmark, Seattle, WA, USA
Rayaz Malik, Manchester, UK
Brady Matthew, Chicago, IL, USA
Vincent Monnier, Cleveland, OH, USA
Irina Obrosova, Ann Arbor, MI, USA
Paolo Pozzili, Rome, Italy
L. Robert, Paris, France
Alexander Rabinovich, Edmonton, Canada
Robert Schmidt, St. Louis, MO, USA
James Sowers, Brooklyn, NY, USA
Martin Stevens, Ann Arbor, MI, USA
Goran Sundquist, Malmo, Sweden
Isabel Valverde, Madrid, Spain
Helen Vlassara, New York, NY, USA
Mark Yorek, Iowa City, IA, USA
Dan Ziegler, Dusseldorf, Germany

				

